# Genes Involved in Feed Efficiency Identified in a Meta-Analysis of Rumen Tissue from Two Populations of Beef Steers

**DOI:** 10.3390/ani12121514

**Published:** 2022-06-10

**Authors:** Amanda K. Lindholm-Perry, Allison M. Meyer, Rebecca J. Kern-Lunbery, Hannah C. Cunningham-Hollinger, Taran H. Funk, Brittney N. Keel

**Affiliations:** 1 Meat Animal Research Center, USDA, ARS, U.S. Clay Center, NE 68933, USA; taran.funk@usda.gov (T.H.F.); brittney.keel@usda.gov (B.N.K.); 2 Division of Animal Sciences, University of Missouri, Columbia, MO 65211, USA; meyerall@missouri.edu; 3 Ward Laboratories, Inc., Kearney, NE 68848, USA; rkern@wardlab.com; 4 Department of Animal Science, University of Wyoming, Laramie, WY 82071, USA; hcunnin6@uwyo.edu

**Keywords:** beef cattle, gene expression, notch signaling, rumen, transcriptome, meta-analysis

## Abstract

**Simple Summary:**

The rumen makes up a large portion of the digestive tract of beef cattle and is responsible for the absorption of nutrients and microbial by-products. The rumen papillae interact with feed, microbial populations, and fermentation products important to cattle nutrition. Variation in the animal’s ability to take up and utilize these nutrients affects feed efficiency. This study was performed to identify genes involved in feed efficiency that are expressed in two unrelated and physically distant populations of Angus and Hereford crossbred steers. A total of 83 genes were identified that may be useful indicators of feed efficiency in cattle. Differentially expressed genes were involved in a protein turnover pathway and a stomach lining turnover pathway. The use of meta-analysis for the two populations of cattle with different sire lines, management and handling techniques, and feed ingredients should allow the identification of genes that are involved in feed efficiency across cattle populations rather than those identified in a single population.

**Abstract:**

In cattle, the rumen is an important site for the absorption of feed by-products released by bacterial fermentation, and variation in ruminal function plays a role in cattle feed efficiency. Studies evaluating gene expression in the rumen tissue have been performed prior to this. However, validating the expression of genes identified in additional cattle populations has been challenging. The purpose of this study was to perform a meta-analysis of the ruminal transcriptome of two unrelated populations of animals to identify genes that are involved in feed efficiency across populations. RNA-seq data from animals with high and low residual feed intake (RFI) from a United States population of cattle (eight high and eight low RFI) and a Canadian population of cattle (nine high and nine low RFI) were analyzed for differences in gene expression. A total of 83 differentially expressed genes were identified. Some of these genes have been previously identified in other feed efficiency studies. These genes included *ATP6AP1*, *BAG6*, *RHOG*, and *YPEL3*. Differentially expressed genes involved in the Notch signaling pathway and in protein turnover were also identified. This study, combining two unrelated populations of cattle in a meta-analysis, produced several candidate genes for feed efficiency that may be more robust indicators of feed efficiency than those identified from single populations of animals.

## 1. Introduction

The feed that enters the rumen is first utilized by microbial communities. These microbes break down the feed into fermentation products that can be transported into the rumen tissue or get passed on through the gut. Variation in microbial populations of the rumen have been shown to affect the feed efficiency of beef cattle [1,2,3]. However, feed efficient cattle may also have variation in their ability to transport or utilize the microbial by-products. Currently, there is only one study evaluating the rumen papillae transcriptome of beef cattle with variation in residual feed intake (RFI), a measure of feed efficiency [4]. Other studies evaluating the transcriptome of rumen papillae tissue with feed intake and gain [5] or evaluating specific candidate genes [6] exist, but it is challenging to compare studies based on different methodologies or phenotypes.

There are few examples of transcriptome meta-analyses with two or more unrelated populations or cohorts of beef cattle [7,8]. These types of studies should provide the identification of genes that underly the phenotype of interest as they account for some of the potentially confounding issues that may influence gene expression in single population studies, such as season, management, and sire lines. Genes that are identified in multi-population studies may produce more robust biological markers for feed efficiency. The purpose of this study was to identify differentially expressed genes in the rumen tissue of a Canadian and a United States population of Angus and Hereford crossbred beef cattle with variation in RFI. 

## 2. Materials and Methods

### 2.1. Cattle Populations

The animal studies were both approved by Institutional Animal Care and Use Committees for livestock research at their respective universities. Details regarding the animals and sample collection from the Canadian population of Hereford × Angus animals can be found in [4]. The data from the Canadian population of animals used in this study was downloaded from the publicly available NCBI Gene expression omnibus database using the series accession number GSE76501. Rumen tissue was collected from the most efficient (*n* = 9; RFI = −1.4 to −2.33 kg/day) and least efficient steers (*n* = 9; RFI = 1.32 to 3.23 kg/day) [4].

The United States population of Angus × Hereford steers with high and low RFI were selected from a larger population of 59 animals. Initial body weights at the start of the study were 461 ± 4.5 kg and initial ages were 379 ± 1.5 days. Animals were fed a high corn finishing diet of 84.8% whole shelled corn, 5.1% alfalfa hay, 6.7% alfalfa haylage, 3.4% protein and micronutrient supplement (11.4% CP, 2.0 Mcal NEm/kg, 1.34 Mcal NEg/kg). Individual feed intakes were measured using the GrowSafe system (model 4000E, GrowSafe Systems Ltd., Airdrie, AB, Canada). Feed intake was monitored for 57 d. Residual feed intake is the difference between the actual and expected feed intake and was calculated by actual dry matter feed intake regressed on average daily gain (ADG) and metabolic midweight (MBW) to determine expected dry matter intake (DMI). Average daily gain was calculated from initial and final steer body weights. At the end of the trial period for the United States population, the most efficient (*n* = 8, low RFI = −0.93 to −1.93) and least efficient (*n* = 8/year, high RFI = 0.89–1.98) steers were selected for harvest. Residual feed intake for both studies was calculated according to Kong et al. [4], using the following model:

(1) $$Y_{j}=\beta_{0}+\beta_{1}MBW_{j}+\beta_{2}ADG_{j}+e_{j}$$

where $Y_{j}$ is the standardized DMI of the *j*th animal, $\beta_{0}$ is the regression intercept, $\beta_{1}$ is the regression coefficient on *MBW*, $\beta_{2}$ is the regression coefficient on *ADG*, and $e_{j}$ is the uncontrolled error of the *j*th animal.

### 2.2. Sample Preparation

Sample preparation of the rumen tissue from the Canadian population of animals was previously described in Kong et al. [4]. Briefly, papillae were collected from the central region of the ventral sac at slaughter and immediately frozen in liquid nitrogen. RNA was isolated with mirVana kit (Ambion, Austin, TX, USA) according to the manufacturer’s instructions. Samples were evaluated on the Agilent 2100 Bioanalyzer and a RIN of >7 was required for sequencing. 

For the United States population of animal samples, rumen papillae tissue from the ventral portion of the cranial sac was rinsed with PBS and flash frozen at the time of harvest. Tissues were stored at −80 °C until RNA isolation. Total RNA was isolated from the rumen tissue using the RNeasy Mini Plus kit and QiaShredder columns (Qiagen, Valencia, CA, USA). Briefly, 800 µL of RLT (lysis) buffer with β-mercaptoethanol were added to 30–50 mg of rumen papillae tissue and homogenized for 40 s using an Omni Prep 6-station homogenizer (Omni International, Kennesaw, GA, USA). The homogenate was centrifuged in a QiaShredder column at 17,000× *g* for 3 min at room temperature. The RNeasy Mini Plus kit manufacturer’s protocol was then followed, and the total RNA was eluted in 50 µL of RNase free water. Total RNA was quantified with a NanoDrop One spectrophotometer (ThermoFisher Scientific, Waltham, MA, USA) and evaluated for quality using an Agilent 2200 TapeStation using RNA ScreenTape and reagents. All RIN values were >8.0.

### 2.3. RNA-Sequencing

Sequencing information for the Canadian population of animal samples was previously described by Kong et al. [4]. Briefly, 18 libraries were generated using the TruSeq RNA Sample Preparation v2 kit (Illumina, San Diego, CA, USA). Libraries were pooled and sequenced on the Illumina HiSeq 2000 system. 

For the United States population, each RNA sample (250 ng) from the 16 steers was prepared for RNA sequencing with the Illumina TruSeq stranded mRNA library preparation kit following the manufacturer’s protocol (Illumina Inc., San Diego, CA, USA). The libraries were quantified with RT-qPCR using the NEBNext Library Quant Kit (New England Biolabs, Inc., Beverly, MA, USA) on a CFX384 thermal cycler (Bio-Rad, Hercules, CA, USA). The size and quality of the library were evaluated with an Agilent Bioanalyzer DNA 1000 kit (Santa Clara, CA, USA). The libraries were diluted to 4 nM with Illumina RSB. Libraries were paired-end sequenced with 150 cycle high output sequencing kits on an Illumina NextSeq 500 instrument.

### 2.4. Data Analysis

Data from each of the two populations was processed using a single pipeline. The quality of the raw paired-end sequence reads in individual fastq files was assessed using FastQC (Version 0.11.5; www.bioinformatics.babraham.ac.uk/projects/fastqc) (accessed on 15 April 2022), and reads were trimmed to remove adapter sequences and low-quality bases using the Trimmomatic software (Version 0.35) [9]. The remaining reads were mapped to the ARS-UCD1.2 genome assembly (NCBI Refseq Accession GCF_002263795.1) using Hisat2 (Version 2.1.0) [10]. The NCBI annotation for ARS-UCD1.2 (Release 106) was used to guide the alignment. Stringtie (Version 1.3.3) [11] was used to determine read counts for each of the 34,624 annotated genes in the ARS-UCD1.2 genome assembly. The raw sequencing data for the U.S. population can be accessed at the NCBI Sequence Read Archive (SRA) database with accession number PRJNA762307.

Meta-analysis of gene expression was conducted by combining *p*-values from per-study differential analyses. For each study, data were analyzed using the DESeq2 package [12] with the following generalized linear model (GLM):

Y = RFI Group.(2)

In this model, a negative binomial link function is used to consider the explanatory variable RFI Group (Low vs. High).

Per-study raw *p*-values for each gene were combined using Fisher’s method [13]. This approach combines *p*-values from each experiment into one test statistic:

(3) $$X=-2\sum_{s=1}^{S}\ln\left(p_{gs}\right),$$

where $p_{gs}$ denotes the nominal *p*-value obtained from gene g in experiment s, and S is the number of experiments being combined. Under the null hypothesis, the test statistic X follows a $\chi^{2}$ distribution with 2S degrees of freedom. This statistical test provides a meta-*p*-value for each gene, and classical procedures for multiple testing correction can then be applied to control the false discovery rate. The Benjamini–Hochberg method [14] was used to correct for multiple testing. Genes with adjusted meta-*p* ≤ 0.05 were considered statistically significant. This meta-analysis procedure was implemented using the metaRNASeq package in R (Version 1.0.7). 

One critical underlying assumption for the use of the Fisher test statistic, defined in Equation (3), is that *p*-values for genes in each of the individual studies are uniformly distributed under the null hypothesis. However, in most RNA-Seq data, a peak in *p*-values close to 1 is observed due to the discretization of *p*-values for very low read counts. As proposed by Rau et al. [15], the HTSFilter package in R (Version 1.34.0; [16]) was used to filter weakly expressed genes in each of the studies. Using this approach for gene filtering, it is possible that genes could be filtered from one study, but not the other. As a final filtering step, genes with adjusted meta-*p* ≤ 0.05 that were filtered from one study were removed. 

Principal component analysis (PCA) was performed using the PCAtools package in R (Version 2.6.0). PCA analysis was performed on normalized gene expression values, generated by DESeq2, removing the lower 10% of genes based on variance. 

The Database for Annotation, Visualization, and Integrated Discovery (DAVID, Version 6.8) [17] was used to identify significantly enriched biological process, molecular function, and cellular component gene ontology terms (GO terms) in the differentially expressed genes. Pathway analysis was performed using the iPathwayGuide software (Version 2201; https://advaitabio.com) (accessed on 20 April 2022). 

## 3. Results

**Figure 1:**
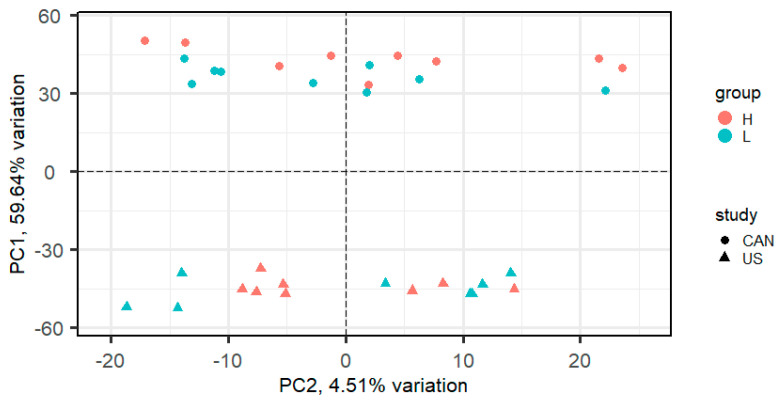
Principal component analysis (PCA) plot of normalized gene expression values in steers with high and low residual feed intake from a combination of a Canadian population and a United States population.

**Figure 2:**
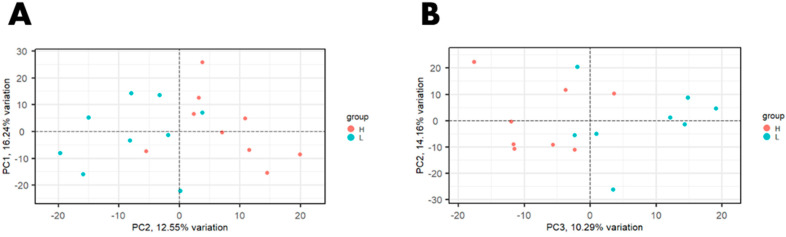
Principal component analysis (PCA) plot of normalized gene expression values in steers with high and low residual feed intake from (**A**) a Canadian population and (**B**) a United States population.

An average of 97.6 million reads per library (*n* = 18) was generated for the Canadian population of beef cattle and an average of 42.6 million reads per library was obtained from the United States population of beef cattle (*n* = 16). Averages of 84.56% and 75.39% of the reads aligned to the ARS_UCD1.2 bovine genome assembly, for the U.S. and Canadian populations, respectively. A total of 14,194 genes were expressed in at least one study. PCA on normalized gene expression values from data across both studies showed that the study (U.S. vs. Canadian study) accounted for the greatest variation (59.64%, PC1; Figure 1). PCA performed on each study individually showed the separation of samples by RFI phenotype (Figure 2). 

**Figure 3:**
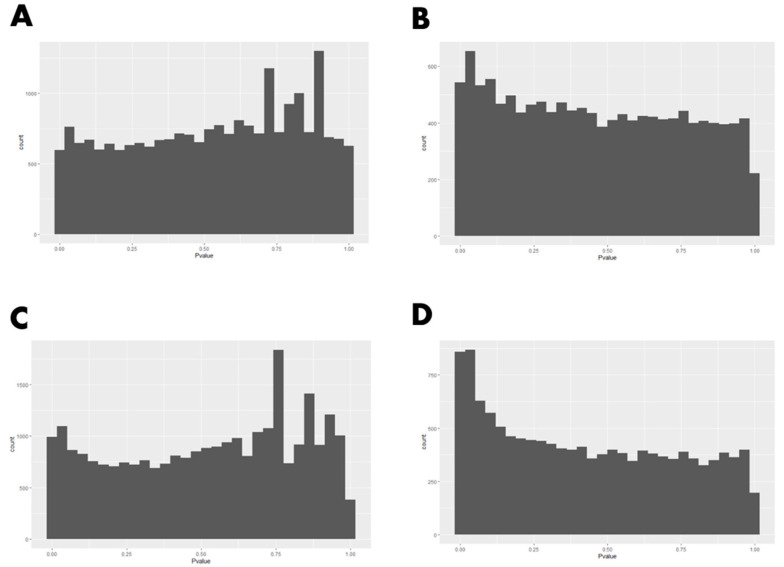
Raw *p*-value histograms for before and after filtering genes using HTSFilter package. (**A**) U.S. data set before filtering; (**B**) U.S. data set after filtering; (**C**) Canadian data set before filtering; (**D**) Canadian data set after filtering.

As part of the meta-analysis procedure, differential gene expression analysis was first conducted for each study (USA and Canadian studies) individually using DESeq2 (Table S1A,B). The HTSFilter package was used to filter lowly expressed genes in each of the studies, resulting in distributions of raw *p*-values that appear to satisfy the uniformity assumption under the null hypothesis that is required for Fisher’s method (Figure 3). Raw *p*-values from these analyses were combined using Fisher’s method to identify differentially expressed genes (DEG) for low RFI versus high RFI animals (Table S1C). A total of 83 DEG were identified (*P*_FDR_ < 0.05; Table 1). 

The robustness of results was assessed by examining the results of the per-study differential expression analyses. Genes were considered robust if they were found to be DEG in both or neither of the individual studies. This is the two-study equivalent to the jackknife sensitivity test described in [8]. There were 12 and 119 DEG identified in the U.S. and Canadian studies, respectively (Table S1A,B). Twenty of the DEG from the meta-analysis were robust enough to pass the jackknife sensitivity test. The robust DEG, *LYPD2*, *ASB2*, *VARS*, *CYP1B1*, *ALPK1*, *B3GNT3*, *C1QBP*, *IL1RN*, *TUBB*, *NDUFA9*, *C7H5orf46*, *FRK*, *REXO5*, *LOC104973218*, *ARAF*, *FAM107B*, *LOC100139345*, *RWDD3*, *SNX15*, and *S100A11,* were not significant in either of the individual studies. The DEG *KAT2B* was the only gene with the same direction of expression that was significant at *P*_ADJ_ ≤ 0.1 in both populations of animals. Figure S1 is a Venn diagram of the genes identified in each of the analyses.

**Figure 4:**
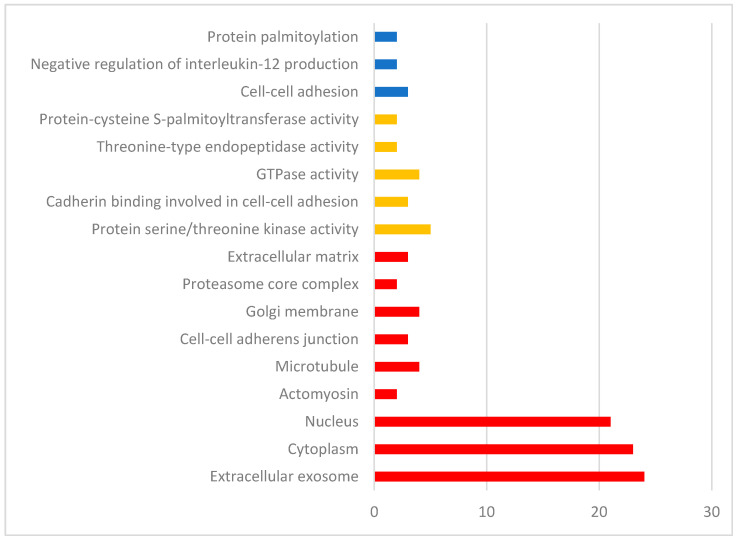
Biological processes (blue), molecular functions (yellow), and cellular components (red) identified by DAVID for the list of differentially expressed genes from the rumen tissue of two populations of steers with high and low RFI.

**Figure 5:**
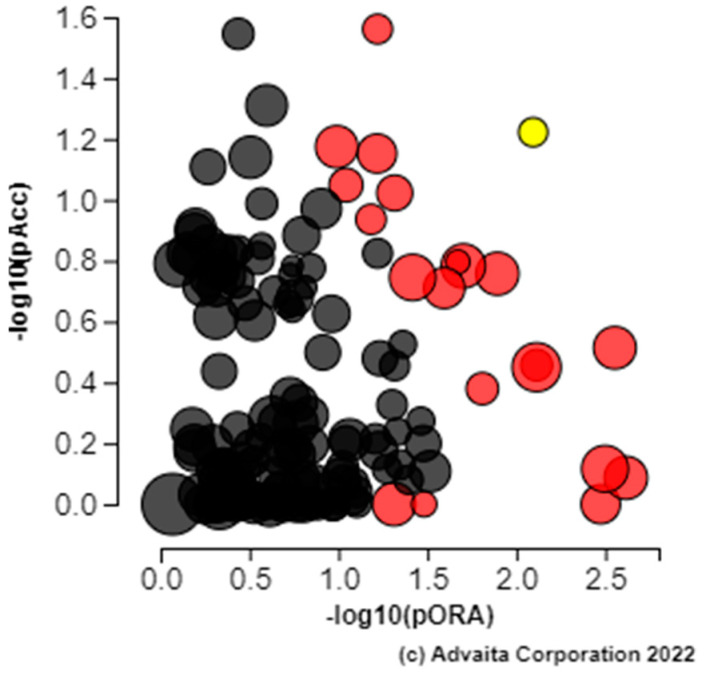
Pathways identified by iPathway Guide as over-expressed from the list of differentially expressed genes from the rumen tissue of two populations of steers with high and low RFI. Renal cell carcinoma (yellow) is shown, using negative log of the accumulation and over-representation *p*-values, along with the other most significant pathways. Pathways in red are significant based on the combined uncorrected *p*-values, whereas the ones in black are non-significant (where applicable).

Gene function analysis was performed using DAVID (Figure 4). The three biological processes identified were cell–cell adhesion and negative regulation of interleukin-12 production (Fisher *P*_ADJ_ < 0.05). Molecular functions included protein serine/threonine kinase activity, cadherin binding involved in cell–cell adhesion, and GTPase activity (*p* < 0.05). Pathway analysis was performed iPathway Guide (Figure 5, Table 2). iPathway Guide identified 19 pathways over-represented in the list of DEG (Fisher *P*_ADJ_ < 0.05). 

## 4. Discussion

This study is the first to integrate RNA-sequencing data from the rumen tissue of two populations of animals from different countries with RFI phenotypes in a meta-analysis. RNA-Seq experiments, especially those performed in livestock, are routinely performed on a small number of biological replicates due to the cost of library preparation and sequencing. The limited power in these studies coupled with both technical and biological variability between studies can lead to issues with reproducibility and cross-validation. Integrating data across multiple experiments may enable the extraction of deeper biological insights compared to that achieved through single-study analysis. 

A study by Rau et al. [15] compared procedures for combining RNA-Seq data from multiple related studies, including *p*-value combination techniques and a negative binomial generalized linear model (GLM) with fixed study effect. The GLM with fixed study effect performed well when inter-study variability was low but was outperformed by *p*-value combination for moderate to large inter-study variability. PCA analysis of the combined USA and Canadian populations (Figure 1) indicated a moderate level of inter-study variability, with PC1 explaining 59.64% of the variation. For this reason, we chose to utilize Fisher *p*-value combination in our meta-analysis.

As previously stated, an underlying assumption of Fisher’s method is that *p*-values are uniformly distributed under the null hypothesis. We note that this was not the case for either the U.S. or Canadian studies (Figure 3A,C), as we see peaks of *p*-values near 1, which results from the discretization of *p*-values for lowly expressed genes. To remove lowly expressed genes, contributing to these peaks, we used the approach proposed by Rau et al. [16]. Removing genes with the HTSFilter package resulted in distributions of raw *p*-values that appear to satisfy the uniformity assumption under the null hypothesis (Figure 3B,D).

The purpose of this study was to identify genes differentially expressed in the rumen of beef cattle associated with RFI that will be robust across the cattle industry for identification or selection of more feed efficient animals. Some of the genes identified were previously reported as differentially expressed in the Canadian population of animals [4]. These genes included *ATP6AP1*, *RHOG*, *S100A11*, *PSMB6*, *UACA*, *YPEL3*, *ZDHHC5*, and *TMEM54* among others. Discrepancies between the DEG found in the Canadian population in this study and those reported in [4] were due to differences in technical protocol, including mapping software (Tophat2 vs. Hisat2) and genome build (UMD3.1 vs. ARS-UCD1.2). This is the first report of the RNA-sequencing data from the United States population of cattle. Thus, no prior differentially expressed genes have been published.

The variation in gene expression values attributed to the study effect (Figure 1) underscores the importance of including animals from more than one study to obtain biologically relevant data for complex traits. Validation of transcriptomic or proteomic data is likely to produce poor reproducibility from study to study due to the large amount of biological variation from sources that include breed, management, and environmental factors. Integration of data across multiple studies, via meta-analysis, can help identify more robust differentially expressed genes.

Genes passing the jackknife sensitivity analysis, i.e., genes that were differentially expressed in both or neither of the individual study analyses, can be considered highly robust, as they are not dependent on just one study. Twenty of the DEG passed the jackknife tests. Genes being driven by a single study in the meta-analysis (i.e., those failing the jackknife test) represent potential false-positives or those whose differential expression is driven by differences in environment or management. The addition of more studies to the meta-analysis should efficiently remove those that are false findings by increasing the number of large *p*-values in the multiplication performed in Fisher’s method, which will increase the meta *p*-value.

There were 37 of the 83 DEG that were concordant in their direction of expression in both populations of animals. Among these genes were *PRR5*, *SESN3*, *PSMB5,* and *PSMB6* involved in TORC2 signaling and proteasomal ubiquitin-independent protein catabolic biological processes. Protein turnover via mTOR and ubiquitin-proteosome pathways have been previously identified as mechanisms involved in RFI in the rumen tissue of beef cattle [18]. That these genes were identified in this study and displayed the same direction of expression, with *PRR5*, *PSMB5,* and *PSMB6* up-regulated and *SESN3* down-regulated among the more feed efficient animals in both populations, suggests that these genes may warrant further investigation into their functional roles in feed efficiency. 

The *KAT2B* gene is the only gene that was significant in both populations after correction for multiple testing at *p* < 0.1 and also displayed the same direction of expression in both populations. The KAT2B protein plays a role in the Notch signaling pathway. Notch cell signaling occurs intercellularly between cells and is involved in apoptosis, cell differentiation, and promoting and suppressing cell proliferation [19]. Widely recognized for its role in early development, Notch signaling is also involved in the self-renewal of adult tissues [19]. A previous study [20] established a role for notch signaling in the foregut of mammals. In adult mice, the gastric epithelium or mucosa continually turns over and renews the cells exposed to the stomach. Kim and Shivdasani [20] showed that notch activation converts stomach epithelial cells into stem or multipotential progenitors that repopulate the mucosa with all major cell types. Over-expression or continued activation of notch signaling can allow the formation of dysplastic adenomas from the de-differentiated progenitors, and the under-expression of notch impairs epithelial proliferation. Furthermore, Obata et al. identified Notch signaling to be key to intestinal immune homeostasis in mice [21]. The pathway was identified as being involved in maintenance of cell turnover and the interaction of colonic epithelium with microbes [21]. A study conducted on lactating Holstein cows showed intentional interruption of the Notch signaling pathway to reduce intestinal barrier function resulted in decreased feed intake and milk yield [22]. While this pathway was not identified in the meta-analysis, the gene *KAT2B*, which is associated with this pathway, was identified as a DEG in both populations, and its direction of expression was the same in both. The gene *KAT2B* was down-regulated in the rumen of more feed efficient animals, suggesting the reduced turnover of rumen mucosal or papillae cells when compared to the animals that were less efficient. A recent transcriptome study of the liver in Jersey and Holstein cattle selected for RFI was designed to identify genetic variants from expressed genes in RNA-sequencing data. A polymorphism in *KAT2B*, a gene involved in the Notch signaling pathway, was identified that segregated with the same RFI phenotypes in both breeds of cattle [23]. The identification of *KAT2B* genetic variant associated with RFI, along with the correlation of *KAT2B* expression with RFI in this study, suggest that *KAT2B* may be of particular importance for feed efficiency in cattle.

Two additional genes with roles in the Notch signaling pathway were also identified. These were *APH1B* (*p* < 0.05) and *BAG6*, which displayed a trend toward significance (*p* < 0.1). The *APH1B* gene encodes one of the four subunits of the γ-secretase complex. APH1B plays an important role in the Notch signaling pathway because it stabilizes the complex [24]. This study appears to be the first report of the involvement of *APH1B* in feed efficiency in livestock. The *BAG6* is involved in the Wnt/Hedgehog/Notch pathway according to the PathCards pathway unification database. Both BAG6 and KAT2B form complexes with p300, a histone acetyltransferase. KAT2B competes with E1A for p300 binding. When KAT2B binds to p300, the complex activates transcription. BAG6 binds to the E1A/p300 complex and controls apoptosis in response to DNA damage. DNA damage can occur because of intracellular metabolism, replication, or oxidative phosphorylation. Metabolic reactions can generate aldehydes or alkylating agents that cause DNA adducts, and oxidative phosphorylation generates reactive oxygen species that can create oxidative DNA damage. The upregulation of *BAG6* among the more efficient animals might suggest that they are better able to react to cellular damage. 

The involvement of oxidative phosphorylation in feed efficiency has been reported in many studies in swine, beef cattle and chickens [25,26,27,28,29]. The study presented here identified a gene involved in the electron transport chain, *NDUFA9*. This gene was nominally significant and up-regulated among the feed efficient animals in both populations of steers. Another study [30] identified two genes (*UQCR10* and *NDUFB4*, *p* ≤ 0.07) involved in oxidative phosphorylation up-regulated in the rumen tissue of bulls with high RFI. The prior study by Kong et al. [4] used a weighted gene co-expression network analysis for the Canadian steers to identify genes involved in oxidated phosphorylation that were up-regulated in the low RFI animals. Increased oxidative phosphorylation has also been reported in the liver and muscle tissue of more efficient cattle [30,31]. It has been proposed that the rumen tissue of more feed efficient animals has higher mitochondrial activity [4], which could support the need for increased response to DNA damage.

The gene *RHOG* was also identified as differentially expressed in this study. The expression of *RHOG* has been previously associated in the rumen tissue of beef cattle with variation in body weight gain making it a compelling candidate for feed efficiency [32,33]. *RHOG* is located in a region of BTA15 that was associated with average daily gain (ADG) in a GWAS on a large population of beef cattle from USMARC [32]. We have also previously shown that *RHOG* expression was positively associated with ADG in two cohorts of beef cattle [33]. RHOG is a small GTPase that functions as a molecular regulator involved in signal transduction cascades and is part of a positive feedback loop for PI3K activation [34], which is part of the Akt/mTOR signaling pathway. The Akt/mTOR signaling pathway is a “master regulator” for protein synthesis [35]. Activated PI3K results in cell signaling for protein synthesis. Previous work in pigs has shown that there is less protein degradation in animals selected for low RFI and it was suggested that this may be a contributing factor to the increased efficiency of these animals [36]. 

## 5. Conclusions

The meta-analysis of the rumen transcriptome of two unrelated and geographically distant populations of Angus and Hereford crossbred steers in this study detected several genes that may be involved in RFI. The identification of genes with a role in the Notch signaling pathway (*APH1B*, *BAG6*, and *KAT2B*), protein turnover (*PRR5*, *SESN3*, *PSMB5*, and *PSMB6*), and those that have been identified previously in other studies (*RHOG*, *ATP6AP1*, and *YPEL3*) provides support for this pathway’s involvement in cattle feed efficiency.

## Figures and Tables

**Table 1:** Genes identified as differentially expressed between low versus high RFI beef cattle in a meta-analysis of two unrelated populations of steers.

Gene Symbol	*P*_Nominal_	*P*_FDR_	Consistent LFC ^1^
*LOC789569*	6.71 × 10^−9^	9.52 × 10^−5^	N
*LOC101904916*	5.48 × 10^−8^	0.000389	Y
*TECR*	1.44 × 10^−7^	0.000680	Y
*ATP6AP1*	2.96 × 10^−7^	0.000788	N
*PAMR1*	3.11 × 10^−7^	0.000788	N
*EGLN3*	7.51 × 10^−7^	0.00120	N
*LOC100848775*	7.60 × 10^−7^	0.00120	N
*LYPD3*	5.98 × 10^−7^	0.00120	N
*KAT2B*	2.46 × 10^−6^	0.00234	Y
*KLK13*	2.22 × 10^−6^	0.00234	N
*PLP2*	2.43 × 10^−6^	0.00234	N
*HTRA1*	2.76 × 10^−6^	0.00245	N
*RHOG*	3.97 × 10^−6^	0.00313	N
*TUBA4A*	3.81 × 10^−6^	0.00313	Y
*CD52*	7.06 × 10^−6^	0.00501	Y
*SH3BGRL3*	7.81 × 10^−6^	0.00528	N
*SESN3*	1.02 × 10^−5^	0.00636	Y
*ZDHHC5*	1.07 × 10^−5^	0.00636	Y
*ZNF750*	1.07 × 10^−5^	0.00636	N
*RPS15*	1.85 × 10^−5^	0.0101	Y
*ODF2L*	2.70 × 10^−5^	0.0137	Y
*SH3GLB2*	2.81 × 10^−5^	0.0138	Y
*HGS*	3.63 × 10^−5^	0.0172	Y
*MYL12A*	4.15 × 10^−5^	0.0190	Y
*ZDHHC3*	4.57 × 10^−5^	0.0203	N
*ASB3*	4.79 × 10^−5^	0.0205	Y
*MYADM*	4.90 × 10^−5^	0.0205	Y
*LOC104976804*	5.56 × 10^−5^	0.0219	Y
*LYPD2*	5.45 × 10^−5^	0.0219	N
*ASB2*	5.90 × 10^−5^	0.0220	N
*CBX2*	5.75 × 10^−5^	0.0220	N
*VARS*	6.67 × 10^−5^	0.0237	Y
*GLULP*	7.17 × 10^−5^	0.0242	Y
*RC3H1*	7.02 × 10^−5^	0.0242	Y
*HSPB1*	7.77 × 10^−5^	0.0251	N
*ZNF146*	7.65 × 10^−5^	0.0251	N
*LY6G6C*	8.04 × 10^−5^	0.0254	N
*CYP1B1*	9.02 × 10^−5^	0.0272	N
*PSMB5*	9.26 × 10^−5^	0.0274	Y
*ALPK1*	9.87 × 10^−5^	0.0277	Y
*DNM2*	9.94 × 10^−5^	0.0277	N
*PSMB6*	9.56 × 10^−5^	0.0277	Y
*B3GNT3*	0.000119	0.0294	N
*C1QBP*	0.000117	0.0294	Y
*NBEAL1*	0.000120	0.0294	Y
*SH3GL1*	0.000110	0.0294	Y
*IL1RN*	0.000130	0.0312	N
*TUBB*	0.000135	0.0314	Y
*SLC35D1*	0.000149	0.0328	N
*TMEM54*	0.000152	0.0331	N
*LOC104971374*	0.000171	0.0357	N
*CCDC66*	0.000178	0.0367	N
*MAN2B1*	0.000189	0.0382	N
*NDUFA9*	0.000194	0.0382	Y
*CFL1*	0.000197	0.0383	Y
*PIBF1*	0.000199	0.0383	N
*C7H5orf46*	0.000208	0.0384	N
*LOC100848030*	0.000206	0.0384	N
*YPEL3*	0.000204	0.0384	N
*MTERF2*	0.000216	0.0392	N
*FRK*	0.000219	0.0394	Y
*ATR*	0.000239	0.0409	N
*REXO5*	0.000238	0.0409	N
*RUVBL1*	0.000251	0.0425	Y
*LOC104973218*	0.000257	0.0425	N
*PRR5*	0.000258	0.0425	Y
*DNAJB1*	0.000265	0.0432	N
*MTAP*	0.000274	0.0438	N
*MAPK1*	0.000278	0.0439	Y
*TMSB10*	0.000298	0.0461	N
*UACA*	0.000298	0.0461	Y
*ARAF*	0.000305	0.0465	N
*DCUN1D4*	0.000317	0.0465	Y
*GABARAP*	0.000315	0.0465	N
*MALL*	0.000318	0.0465	N
*RGS5*	0.000316	0.0465	Y
*FAM107B*	0.000335	0.0476	Y
*LOC100139345*	0.000345	0.0476	N
*PNPT1*	0.000346	0.0476	N
*RWDD3*	0.000334	0.0476	N
*SNX15*	0.000341	0.0476	N
*ELF5*	0.000354	0.0483	Y
*S100A11*	0.000360	0.0486	Y

^1^ Log2 FC (LFC) is considered consistent if the sign of the LFC is the same for both studies.

**Table 2:** Top pathways and their associated *p*-values identified by iPathway Guide as over-expressed from the list of differentially expressed genes from the rumen tissue of two populations of steers with high and low RFI.

Pathway	#DEG ^1^	*P*	Genes
Renal Cell Carcinoma	3	0.004	*ARAF*, *EGLN3*, *MAPK1*
Salmonella Infection	6	0.007	*DNM2*, *MAPK1*, *MYL12A*, *RHOG*, *TUBA4A*, *TUBB*
p53 Signaling Pathway	2	0.012	*ATR*, *SESN3*
Phagosome	4	0.012	*ATP6AP1*, *HGS*, *TUBA4A*, *TUBB*
Prion Disease	6	0.014	*MAPK1*, *NDUFA9*, *PSMB5*, *PSMB6*, *TUBA4A*, *TUBB*
Parkinson Disease	5	0.016	*NDUFA9*, *PSMB5*, *PSMB6*, *TUBA4A*, *TUBB*
Alzheimer Disease	5	0.017	*ARAF*, *MAPK1*, *NDUFA9*, *PSMB5*, *PSMB6*, *TUBA4A*, *TUBB*
Gap Junction	3	0.019	*MAPK1*, *TUBA4A*, *TUBB*
Pathways of Neurodegeneration—Multiple Diseases	7	0.019	*ARAF*, *MAPK1*, *NDUFA9*, *PSMB5*, *PSMB6*, *TUBA4A*, *TUBB*
Huntington Disease	5	0.022	*NDUFA9*, *PSMB5*, *PSMB6*, *TUBA4A*, *TUBB*
Bladder Cancer	2	0.023	*ARAF*, *MAPK1*, *NDUFA9*, *PSMB5*, *PSMB6*, *TUBA4A*, *TUBB*
Axon Guidance	3	0.028	*CFL1*, *MAPK1*, *MYL12A*
Serotonergic Synapse	2	0.029	*ARAF*, *MAPK1*
Regulation of Actin Cytoskeleton	4	0.031	*ARAF*, *CFL1*, *MAPK1*, *MYL12A*
Fc Gamma R-mediated Phagocytosis	3	0.039	*CFL1*, *DNM2*, *MAPK1*
Human T-cell Leukemia Virus 1 Infection	3	0.041	*ATR*, *KAT2B*, *MAPK1*
Amyotrophic Lateral Sclerosis	5	0.041	*NDUFA9*, *PSMB5*, *PSMB6*, *TUBA4A*, *TUBB*
Bacterial Invasion of Epithelial Cells	2	0.045	*DNM2*, *RHOG*
Parathyroid Hormone Synthesis, Secretion and Action	2	0.047	*ARAF*, *MAPK1*

^1^ #DEG: Number of DEG identified in each pathway.

## Data Availability

The raw sequencing data for the steer samples from the Canadian population can be found at the Gene Expression Omnibus under the GEO series accession number GSE76501. The raw sequencing data for the steer samples from the United States population can be accessed at sequence read archive (SRA) database with accession number PRJNA762307.

## Supplementary Materials

The following supporting information can be downloaded at: https://www.mdpi.com/article/10.3390/ani12121514/s1, Supplementary Table S1—(A) US Population (B) Canadian Population (C) Meta-analysis. Figure S1: Venn diagram illustrating the numbers of differentially expressed genes identified in the United States population and Canadian populations of steers, and those identified in the meta-analysis. Overlapping regions of the circles includes the number of genes common to more than one analysis.
