# Supplementary figures and images for: Genes Involved in Feed Efficiency Identified in a Meta-Analysis of Rumen Tissue from Two Populations of Beef Steers
Source: Animals (Basel). 2022 Jun 10;12(12):1514. doi: 10.3390/ani12121514 (PMC9219435; doi:10.3390/ani12121514)

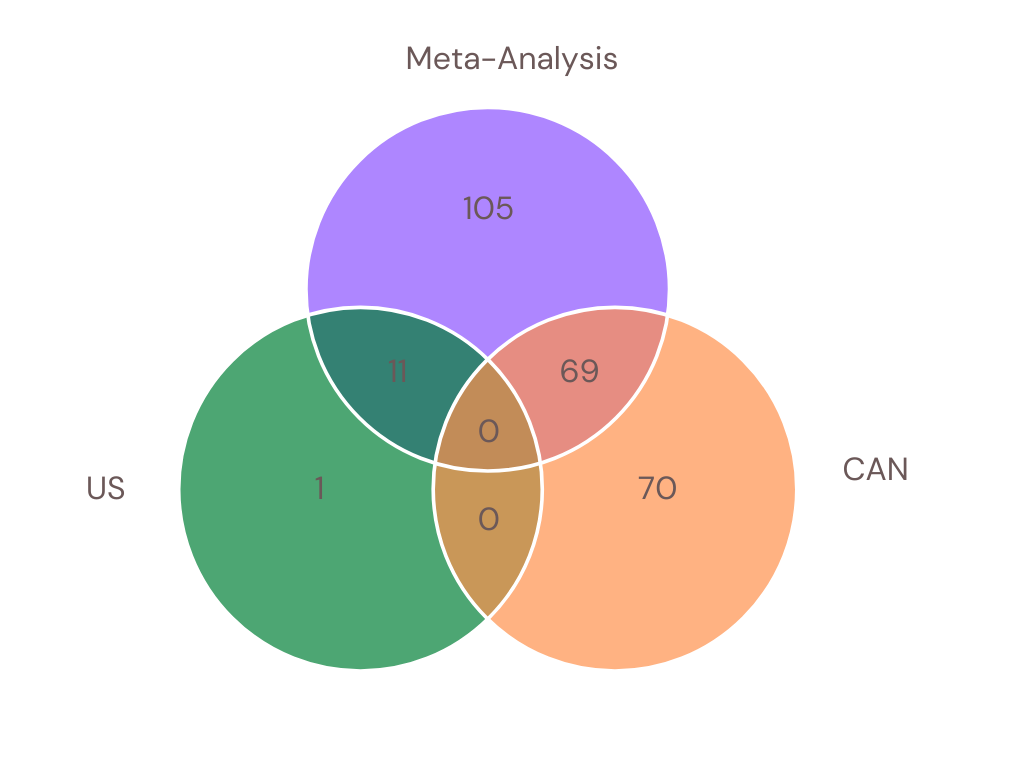

Supplement: Supplementary file 1 [file animals-12-01514-s001.zip › animals-1743035 supplementary/animals-1743035-figure S1.png]
